# Fluidity and Strength of Loess-Based Quick Consolidated Backfill Material with One High-Water Content

**DOI:** 10.3390/ma16165544

**Published:** 2023-08-09

**Authors:** Chenghao Cui, Baifu An, Heng Cui, Qiaomei Yi, Jiale Wang

**Affiliations:** 1 Work Safety Key Laboratory on Prevention and Control of Gas and Roof Disasters for Southern Coal Mines, Hunan University of Science and Technology, Xiangtan 411201, China; 21020101080@mail.hnust.edu.cn (C.C.); 20010101004@mail.hnust.edu.cn (Q.Y.); 22020101013@mail.hnust.edu.cn (J.W.); 2 Shandong Energy Xinwen Mining Group Co., Ltd., Taian 271200, China; liutao@shandong-energy.com; 3 Binxian Shuiliandong Coal Mine Co., Ltd., Binzhou 713500, China

**Keywords:** loess-based, backfill, initial coagulation time, diffusion, compressive strength, shear strength, analysis model

## Abstract

To study the flow and strength characteristics of loess-based backfill materials, orthogonal tests were used to design a cemented backfill material combining loess, high-water content materials, cement, and fly ash. By using the range, analysis of variance, and multi-variate regression analysis, influences of four key factors on the initial setting time, diffusivity, compressive strength, and shear strength of the backfill material were investigated. These four factors included the mass concentration of loess water (A), the content of high-water content materials (B), cement content (C), and content of fly ash (D). The results showed that the initial setting time, diffusivity, compressive strength, and shear strength of the backfill material were 13~33 min, 400~580 mm, 0.917–3.605 MPa, and 0.360–0.722 MPa, respectively, all distributed in wide ranges. For the initial setting time, the four factors were listed in descending order as A > D > B > C according to their influences; for diffusivity, the four factors were listed as A > B > C > D; for the compressive strength, the four factors were ranked as A > C > D > B; for the shear strength, the four factors were ranked such that A > C > D > B. With regard to the comprehensive index, the four factors were such that A > B > D > C. That is, the factors were listed in descending order as the mass concentration of loess water, cement content, the content of fly ash, and content of high-water content materials according to their significance in influencing characteristics of the loess-based backfill material. Comprehensive analysis indicated that the fluidity of the material was mainly influenced by the mass concentration of loess water, and the two were negatively correlated. The hydro-consolidation effect of materials with high-water contents accelerated material solidification. The strength of the backfill material was mainly influenced by the cement content while only slightly affected by contents of other materials. In this way, a prediction model for characteristic parameters, namely, fluidity and strength, of the loess-based backfill material under the action of various factors was established.

## 1. Introduction

Loess, as an important geological material, plays a key role in filling technology. Filling technology involves waste or recyclable materials generated in the process of mining or other underground mining, through reasonable treatment and arrangement, being a backfill to the goaf or pre-mined areas [1,2]. This cannot only solve the geological environment problems remaining as legacy issues after mining, but can also improve the recovery rate of resources to the greatest extent and promote sustainable development [3].

Loess has a series of unique properties, making it an ideal choice for filling materials. Loess is a natural soil composed of fine soil particles, the main components of which are clay minerals and non-clay minerals [4,5]. These components make loess demonstrate good cohesiveness, fluidity, and durability, which is conducive to achieving stable volume growth and uniform distribution in the filling process. In addition, loess also has low permeability and better water stability, which helps to reduce the waste of water resources and the risk of occurrence of geological disasters [6,7,8,9].

With the continuous development of mining activities, filling technology will play a more important role in the future. In mining activities, with the deepening of the mine, the goaf and waste accumulation become more serious, affecting the environment [10,11,12]. By using loess as filling material, not only can the gob be filled, but this can provide better geological stability and support, and reduce the risk of surface subsidence and geological disasters. In addition, as an environmentally friendly filling material, loess will help reduce the damage to the natural ecological environment in the mining process and promote the development of the mining industry in the direction of green and environmental protection [13,14,15].

The present research aims to explore the application potential of loess as a filling material in mining, analyze the properties and characteristics of loess material in depth, and forecast its future application in mining activities. By means of systematic research and discussion, we attempt to provide valuable reference and theoretical support for promoting the development and optimization of mine filling technology, and encouraging the mining industry toward a more sustainable and environmental protection direction [16,17,18,19,20].

## 2. Orthogonal Test Schemes and Test Results

### 2.1. Experimental Raw Material Analysis

By adjusting all factors and levels, orthogonal experimental design can obtain more comprehensive results in fewer experiments and is used to determine the best factor ratio [21].

Firstly, an analysis was conducted on four types of experimental raw materials, namely loess, high-water-content material, cement, and fly ash. The loess was natural loess collected in the Loess Plateau area of Shaanxi Province. The high-water-content material was developed and produced by China University of Mining and Technology. The cement was 42.5 ordinary Portland cement (the C-S-H content was approximately 15% to 20%) and the fly ash was second-grade fly ash produced by the power plant (the CaO content was approximately 8% to 12%.).

The high-water-content material was composed of two materials; high-water-content material A was composed of sulfoaluminate cement clinker, a small amount of ultra-retarding agent, and suspension agent; high-water-content material B was composed of lime, gypsum, a suspension agent, and a composite rapid setting and early strength agent. The two materials do not solidify through pulping alone, and rapidly react after mixing. In the experiment, high-water-content materials A and B each accounted for half of the added amounts of such materials [22,23].

**Figure 1:**
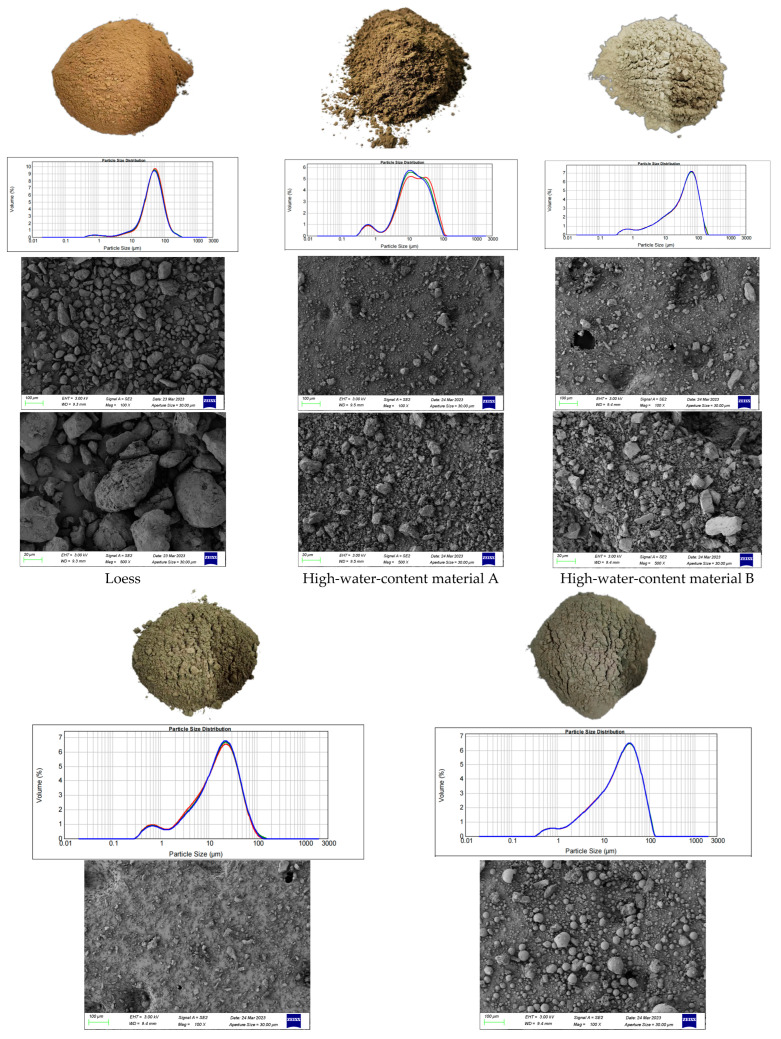

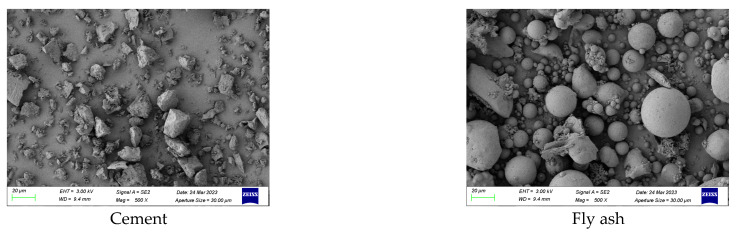
From top to bottom, the image shows the original experimental material, the particle size analysis results, and 100× and 500× magnification SEM micrographs. In a particle size analysis diagram, different colored lines represent multiple measurements of the same material.

The original images of the experimental materials, particle size analysis, and electron microscope scanning are shown in Figure 1.

Particle size analysis: loess was selected as the main filling material, and high-water-content material was added as a water reducer, with cement and fly ash used as the binder. The particle size of the loess material mainly ranged from 10 μm to 500 μm, with a higher proportion of particles in the 10 μm to 100 μm range. The high-water-content material was divided into two types, A and B, with particle sizes mainly distributed from 1 μm to 100 μm. The particle size of the cement material mainly ranged from 1 μm to 100 μm, while the particle size of the fly ash mainly ranged from 1 μm to 100 μm. Due to the varying composition and moisture content of different minerals in the experimental materials, the particle density might be affected. If mass percentages are used, the particle size distribution could be influenced, leading to misleading interpretations. Therefore, in Figure 1, the particle size distribution is presented in volume percentages.

Electron microscopy analysis: by using a scanning electron microscope (SEM) to magnify the experimental material 2000 times, the three-dimensional morphology and topological structure of the sample surface were obtained, and the composition of the material was analyzed.

(1) Loess: the microstructure of the loess mainly presents uniformly sized block shapes, and the clay minerals exhibit a distinct layered structure, imparting strong adsorption performance and plasticity to the soil while the loess has a high specific surface area and porosity, and these pores play an important role in the transportation of water and gases.

(2) High-water-content material: the microstructure of high-water-content material is irregular particles with uneven particle size and rough and irregular surfaces. The rough surface increases its surface area, which is conducive to the adhesion of gels and other substances generated by hydration on its surface, thereby making the bond between the mixture particles closer.

(3) Cement: the microstructure of cement presents irregular block shapes, with small particle sizes and a rough surface. The tiny particles can rapidly enter the pores between larger particles.

(4) Fly ash: the microstructure of fly ash is relatively regular, presenting spherical particles with high density and low surface area. These spherical particles are conducive to their own transportation and flow, and are more likely to pass through the pores between larger particles, thereby increasing the opportunity to react with the hydration products of cement.

Based on the analysis of the experimental materials above, yellow soil, as the main filling material, was combined with some materials to improve the performance of yellow soil. High-water-content material can act as a water reducer, reducing the water content in the filling material, thereby improving the strength and durability of the filling body. Cement and fly ash can be used as binders to increase the strength and hardness of the filling body (all experimental levels were designed in proportion to mass).

### 2.2. Experimental Scheme Design

According to previous experience in preparing paste backfill materials, three materials, namely the high-water-content material, cement, and fly ash, were used as additives to prepare the cemented backfill material together with loess water. Through several simple proportioning tests, the prepared loess-based cemented backfill material was found to be both well consolidated and economically efficient when the mass concentration of loess water was 35%, 40%, 45%, and 50%, and mass fractions of the high-water-content material, cement, and fly ash were 2.5~5.0%, 5.8~9.5%, and 4.1~7.3% of the total, respectively. In the following articles, the mass concentration of yellow mud, high-water-content material, cement content, and fly ash content are all replaced by A, B, C, and D.

Combined with the field backfilling practice in coal mines and for the convenience of description and calculations, the total mass of loess water was fixed at 400 (percentage) in the design, and four test levels for four factors were determined. On this basis, the orthogonal test table and scheme of four factors at four levels were designed, please refer to Table 1 for details.

The fluidity of the material prepared at a room temperature of 25 °C was measured. The samples were placed in a curing box at 25 °C and a relative humidity of 90% to be cured for 28 d. Thereafter, the uniaxial compressive strength and uniaxial shear strength of each sample were measured. The test results are listed in the table below. Equal proportional weights of 0.25 were assigned to four indices, and their sum was the summative quantitative index. It is worth noting that, differently from cement-based backfill materials, loess-based backfill material has such a low strength that its initial setting time cannot be tested using a Vicat apparatus. Therefore, a test method for high-water-content materials was used: the prepared slurry was allowed to stand in a beaker for a period of time and then the beaker was inclined to 45°; if the slurry was consolidated and did not flow, it was regarded as initially set and the corresponding time was noted as the initial setting time.

Table 2 shows that the initial setting time, diffusivity, compressive strength, and shear strength of the loess-based backfill material are, 13~33 min, 0.92~3.61 MPa, 0.92~3.61 MPa, and 0.36~0.72 MPa (all wide ranges), respectively. After the range analysis of the experimental data is detailed in Table 3, by comparing the extreme values R of the four factors, the following conclusions can be drawn. For the initial setting time, the four factors were listed in descending order as D > A > B > C according to their influences; for the diffusivity, the four factors were ranked such that A > D > B > C; for the compressive strength, the four factors were ranked such that A > D > C > B; for the shear strength, the four factors were ranked such that C > D > B > A. As for the comprehensive index, the four factors were ranked such that A > B > D > C. That is to say, the mass concentration of loess water, the content of high-water-content materials, the content of fly ash, and cement content were listed in descending order according to their significance in their influencing characteristics of the loess-based backfill material.

### 2.3. Experimental Procedure

The main testing tools include: a 1000-mL beaker, a thermometer, a hand-held electric mixer, a 70.7 mm × 70.7 mm × 70.7 mm standard three-gang mold, an electronic scale (0.1-g gradations), a YH-60B curing box, and a MTS electro-hydraulic servo-motor-driven machine.

**Figure 2:**
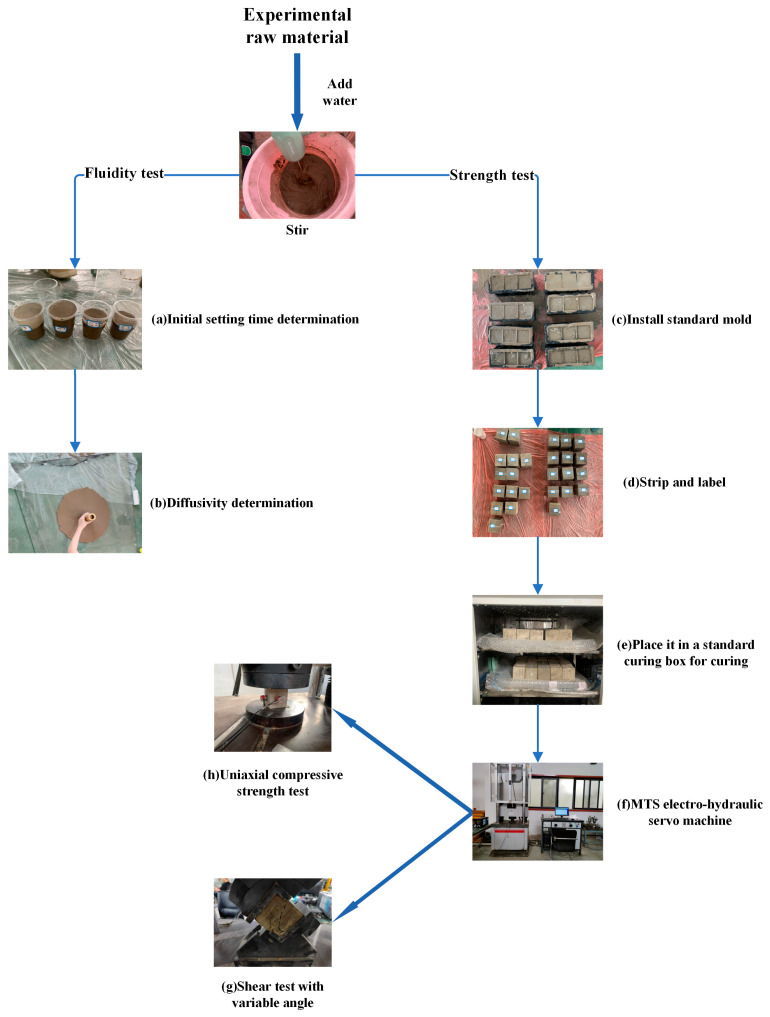
The picture shows a detailed flowchart of the experimental procedure.

The specific testing steps are as follows:

(1) Brush the inner surface of the three-gang mold with lubricating oil before the experiment to ensure that the test piece is relatively intact after demolding;
(2) Weigh the corresponding mass of raw materials according to Table 2, and mix them with water in the mixing bucket;
(3) Pour out some mixed materials and perform initial setting time testing in the beaker. When the beaker is tilted at 45°, there is no obvious flow trace of the material in the cup, which is considered the initial setting of the material (Figure 2a);
(4) Conduct the flow spread test on a smooth glass plate. Apply butter to the bottom of the flow spread cylinder and place it on the glass plate. Pour the mixed filling material into the cylinder to two-thirds of the total height, lift the cylinder and measure the material spread using a tape measure (Figure 2b);
(5) After the material is mixed, it is poured into the standard mold, appropriately vibrated to eliminate air bubbles in the slurry, and stands for three days. Using an air gun to demold the test piece, the demolded specimen is placed in a constant temperature and humidity curing box for curing (Figure 2c,d);
(6) After curing, the specimen is placed on the MTS electro-hydraulic servo-motor machine for the direct shear and uniaxial compressive tests (Figure 2f,g).

## 3. Significance Analysis of Fluidity

### 3.1. Factors Influencing the Initial Setting Time

**Figure 3:**
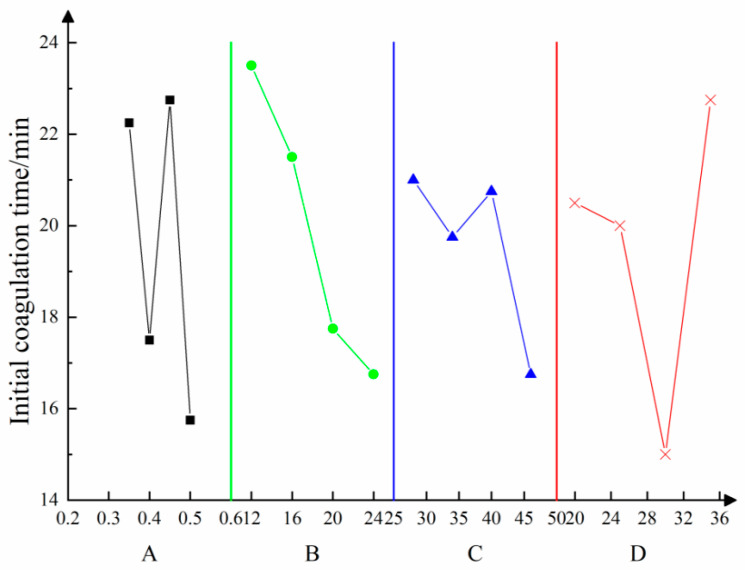
Relationships of the initial setting time with multiple factors—A is the mass concentration of yellow mud; B is for high-water content; C is the cement content; D is fly ash content.

According to the orthogonal test results, the influences of multiple factors on the initial setting time are illustrated in Figure 3. Various factors are shown to have different influences on the initial setting time. According to the analysis of the test results, the content of fly ash is listed in the error column to carry out significance tests at each level (at significance levels *α* of 0.01, 0.05, 0.10, and 0.25). The analysis of variance is summarized in Table 4.

As the initial setting time of the experimental materials is sensitive to various factors, the measurement data in Figure 3 appear to be scattered. To mitigate this variability and improve the accuracy of the results, we conducted multiple rounds of testing for the initial setting time. The data presented represents the average of three separate tests.

An analysis of the results of variance reveals that A and D have some influences, while B and C exert insignificant influences on the initial setting time. The results indicate that the contents of loess and fly ash are the main factors that affect the initial setting rate, while influences of the contents of high-water-content materials and the content of cement are insignificant. In the analysis of variance of the initial setting time, because the variance value of the four factors is too small, i.e., less than twice the error, it is treated as an error term, and the *F* value is not calculated.

### 3.2. Factors Influencing the Diffusivity

**Figure 4:**
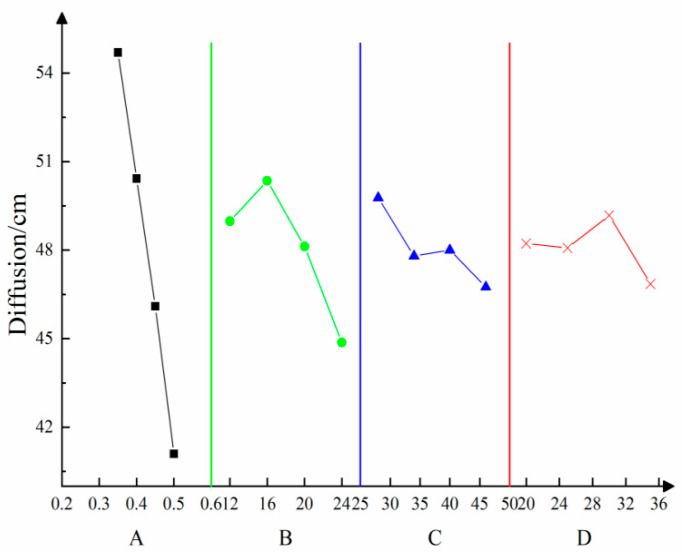
Relationships of diffusivity with multiple factors—A is the mass concentration of yellow mud; B is the high-water content; C is the cement content; D is fly ash content.

The influence of multiple factors on diffusivity was estimated according to the orthogonal test results, as displayed in Figure 4. As shown in the figure, various factors exert different influences on the diffusivity. In accordance with the analysis of the test results, the cement content was listed in the error column to conduct significance tests at various levels. Table 5 summarizes the analysis of variance.

An analysis of the results of variance suggests that A exerts extremely significant influences, B exerts highly significant influences, while C has certain influences, and D does not exert obvious influences on diffusivity. According to the *F* value (*F_D_* > *F_B_*), the mass concentration of loess water exerts more remarkable influences on the content of high-water-content materials when changed at the designed levels. The result indicates that the mass concentration of loess water and the content of high-water-content materials are the main factors influencing diffusivity.

## 4. Significance Analysis of Strength

### 4.1. Factors Influencing the Compressive Strength

**Figure 5:**
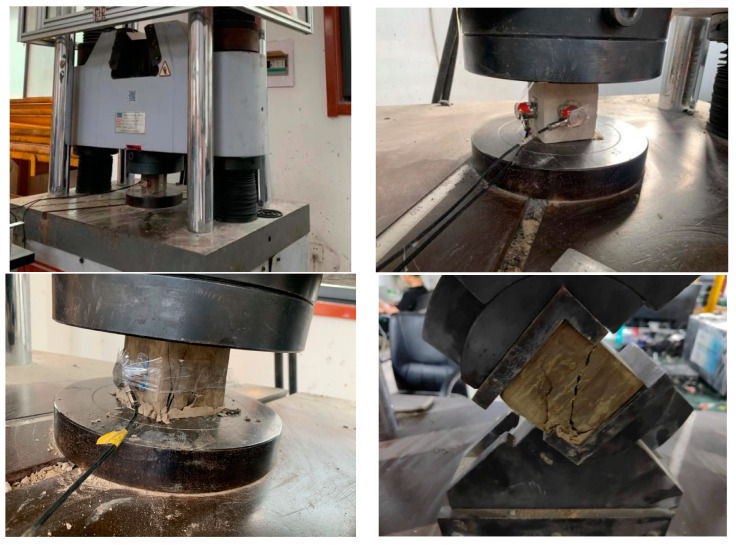
Compressive strength test and some details of the failure modes of loaded specimens.

The MTS electro-hydraulic servo-motor machine was used to perform compressive testing on specimens. Two specimens were taken from each group, and the curing period was 28 days. The test procedure adopted displacement control, and the stress loading rate was set to 0.5 mm/min. The MTS electro-hydraulic servo system measures the real-time changes in stress and strain by connecting to an external computer and utilizing corresponding computer software. The test procedure is shown in Figure 5.

The data collected from the experiment are processed using Equations (1) and (2).

(1) $$\sigma =\frac{F}{l^{2}}$$

(2) $$\varepsilon =\frac{\Delta l}{l}$$

σ—Surface stress on the specimen, MPa.

l—The side length of the standard specimen is 70.7 mm.

Δl—The compression amount of the specimen during the testing process is given in millimeters (mm).

ε—The strain in the specimen.

**Figure 6:**
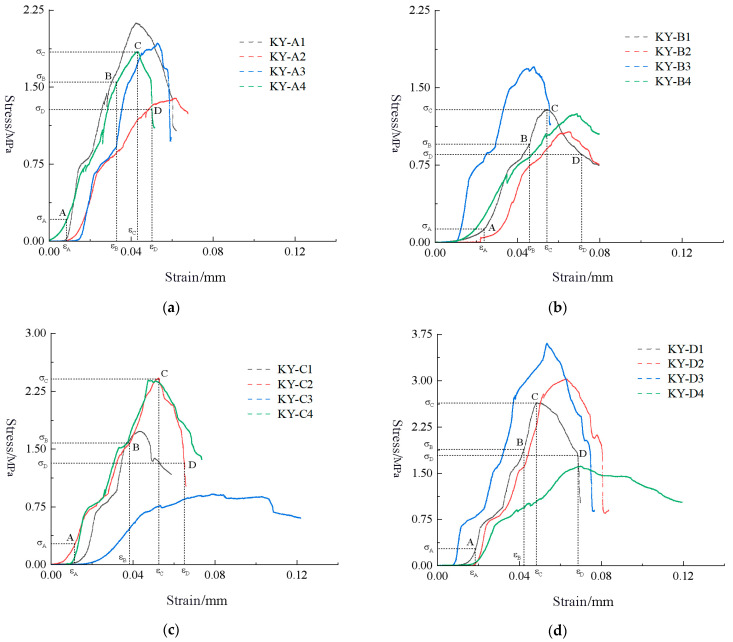
Stress-strain curve. (**a**) KY-A; (**b**) KY-B; (**c**) KY-C; (**d**) KY-D.

The data collected from the experiment are plotted as a stress-strain curve (Figure 6).

**Figure 7:**
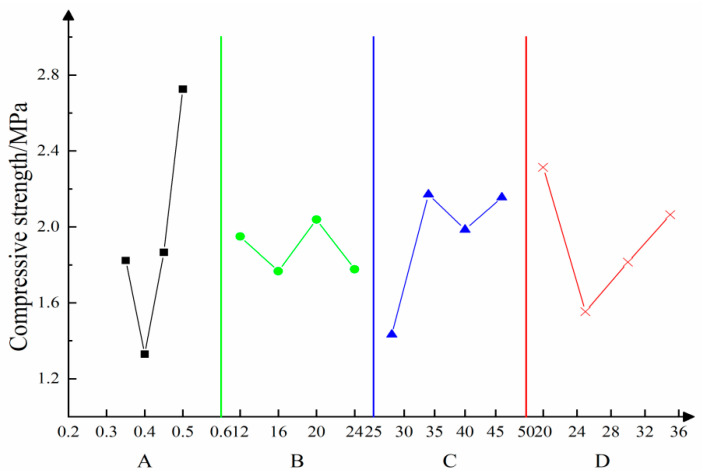
Relationships of the compressive strength with multiple factors—A denotes the mass concentration of yellow mud; B is for high-water content; C is the cement content; D is fly ash content.

Based on the orthogonal test results, the influences of multiple factors on the uniaxial compressive strength are shown in Figure 7; various factors differ in terms of their influences on the compressive strength. According to the analysis of the test results, significance tests were conducted by listing the cement content in the error column. The analysis of variance is summarized in Table 6.

An analysis of the results of variance show that A exerts highly significant influences on the compressive strength. Factors C and D exert weaker influences, and D does not exert obvious influences. The results indicate that the gravimetric moisture content of the loess water is the main factor affecting the uniaxial compressive strength.

### 4.2. Factors Influencing the Shear Strength

**Figure 8:**
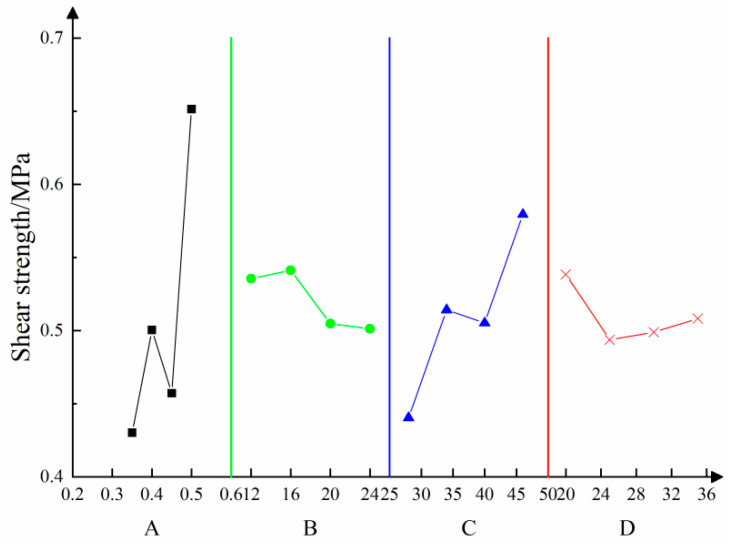
Relationships of the shear strength with multiple factors—A is the mass concentration of yellow mud; B is the high-water content; C is the cement content; D is the fly ash content.

Influences of multiple factors on the uniaxial shear strength were obtained according to the orthogonal test results (Figure 8). Figure 8 shows that the influences of various factors on the shear strength differ. According to the analysis of the test results, the amount of high-water-content materials was listed in the error column when conducting significance tests at various levels. Table 7 summarizes the analysis of variance.

Analysis results of variance indicate that A and C exert extremely significant influences. Factors B and D do not exert significant influences on the shear strength within the given ranges. This shows that the loess content and the cement content are the main factors affecting the shear strength.

## 5. Analytical Model for Characteristic Parameters of Fluidity and Strength

### 5.1. Changes in Characteristic Parameters under Influences of Each Single Factor

**Figure 9:**
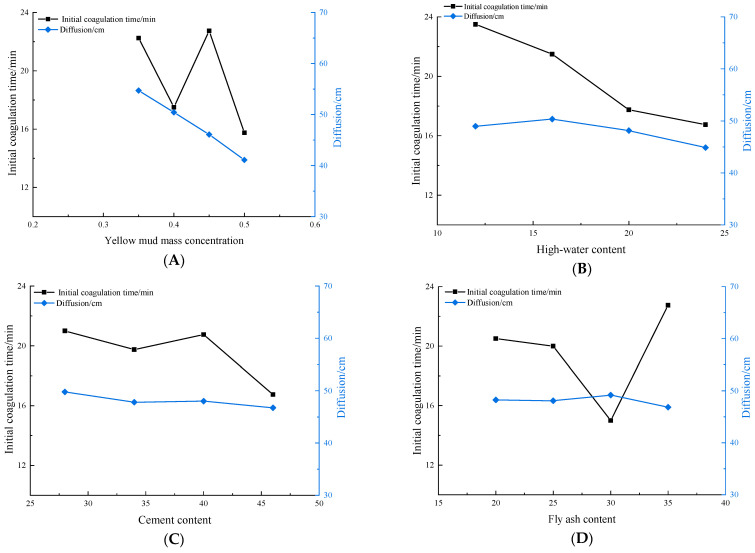
Analysis of material fluidity under the influences of different factors—(**A**–**D**) are the effect curves of yellow mud mass concentration, high-water content, cement content, and fly ash content on initial setting time and diffusion degree, respectively.

By conducting the aforementioned tests at different proportions, the influences of various factors on the fluidity and strength of the backfill material were obtained (Figure 9). It can be seen from the figure that the initial setting time decreased first, increased, and then decreased in A and C, gradually decreasing as B increased, while reducing, then increasing with the increase in D. The diffusivity gradually reduces when A and C increase, first increasing and then decreasing with the increases in B and D. The compressive strength decreased first and then increased later with increases in A and D; it first decreases, then increases and then decreases as B increases, while first increases, then decreases, and then increase as C increases. The shear strength tends to increase at first, then decreases, and then increases as A increases, gradually reducing with the increase in B, gradually increasing with the increase in C, while decreasing, then increasing as D increases.

### 5.2. Analytical Model of Characteristic Parameters

**Figure 10:**
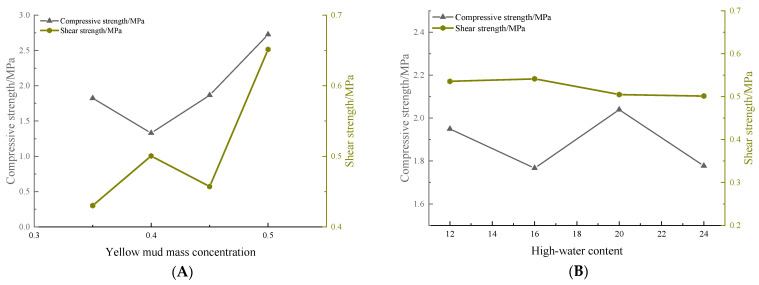

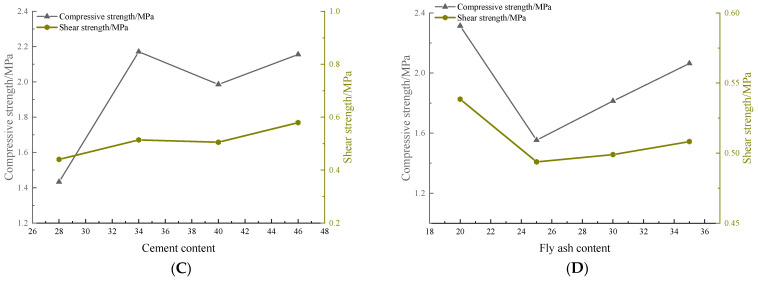
Material strength analysis under the influences of different factors—(**A**–**D**) are the influence curves of yellow mud mass concentration, high-water content, cement content, and fly ash content on compressive strength and shear strength, respectively.

The different influencing factors seen in Figure 9 and Figure 10 were fitted with parametric curves pertaining to similar material. In this way, the relationships of the mass concentrations of loess water, content of high-water content materials, cement content, and content of fly ash with the initial setting time, diffusivity, compressive strength, and shear strength were determined, as shown in Table 8.

**Figure 11:**
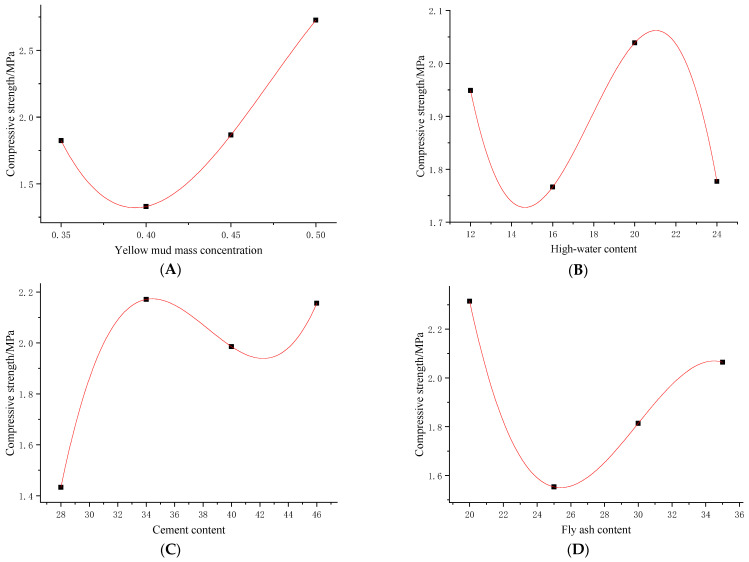
For the compression fitting curves of different factors—(**A**–**D**) are the fitting formula curves of yellow mud mass concentration, high-water content, cement content, and fly ash content, respectively.

As presented in Table 8, *P_A_*, *P_B_*, *P_C_*, and *P_D_* represent the mass concentration of yellow mud, high-water-content material, cement content, and fly ash content, respectively; *t* represents the initial setting time, *K* is the degree of diffusion, *σ_D_* denotes the compressive strength, and *σ_C_* is the shear strength. *R*^2^ represents the goodness of fit of the fitted model to the data. Figure 11 shows the curve of the fitting formula for the compressive strength of the material under the influence of four factors.

The table shows that the initial setting time has a triangular-function relationship with factor A, and has an exponential relationship with factors B and C, while it is in a cubic polynomial relationship with D. The diffusivity is exponentially related to A, while it has a cubic polynomial relationship with B, C, and D. The compressive strength has cubic polynomial relationships with factors A, B, C, and D. The shear strength has cubic polynomial relationships with factors A, B, C, and D.

## 6. Conclusions

(i). By conducting orthogonal tests, taking the mass concentration of loess water, the content of high-water-content materials, the cement content, and the content of fly ash as four factors, for each of which four levels were set, sixteen groups of mix designs were tested. After testing the fluidity and strength characteristics, the analysis revealed that the initial setting time, diffusivity, compressive strength, and shear strength of the loess material were distributed in ranges of 13~33 min, 400~580 mm, 0.917~3.605 MPa, and 0.360~0.722 MPa, respectively;
(ii). For the initial setting time, the four factors were listed in descending order as A > D > B > C, wherein all four factors showed lower significance. The four factors were ranked such that A > B > C > D according to their influences on the diffusivity, indicative of the mass concentration of loess water being the main factor influencing the diffusivity. With regard to the compressive strength, the four factors were ranked such that A > C > D > B, implying that the mass concentration of loess water and the cement content were the main factors influencing the uniaxial compressive strength. The four factors were ranked such that A > C > D > B according to their influences on the shear strength, which means that the mass concentration of loess water and the content of cement were the main factors influencing the shear strength;
(iii). Mathematical relationships of factors A, B, C, and D with the fluidity and strength indices were fitted.

## Figures and Tables

**Table 1:** Orthogonal test factor table (designed by mass scale).

Number of Level Groups	A. Loess Mass Concentration	B. High-Water Content	C. Cement Content	D. Fly Ash Content
1	140:260 (concentration 35.00%)	12	28	20
2	160:240 (concentration 40.00%)	16	34	25
3	180:220 (concentration 45.00%)	20	40	30
4	200:200 (concentration 50.00%)	24	46	35

**Table 2:** The orthogonal test ratio design and experimental results.

Number of Experimental Groups	Influencing Factors				Test Results				
	A	B	C	D	Initial Coagulation Time/min	Diffusion /mm	Compressive Strength/MPa	Shear Strength/MPa	Overall Rating
1	0.35	12	28	20	28.00	58.00	2.13	0.41	22.135
2	0.35	16	34	25	28.00	56.70	1.39	0.43	21.630
3	0.35	20	40	30	16.00	56.30	1.93	0.40	18.658
4	0.35	24	46	35	17.00	47.80	1.85	0.48	16.783
5	0.40	12	34	30	13.00	51.00	1.29	0.51	16.450
6	0.40	16	28	35	23.00	53.70	1.07	0.41	19.545
7	0.40	20	46	20	17.00	49.30	1.71	0.62	17.158
8	0.40	24	40	25	17.00	47.70	1.25	0.46	16.603
9	0.45	12	40	35	33.00	45.70	1.73	0.50	20.233
10	0.45	16	46	30	18.00	48.70	2.42	0.50	17.405
11	0.45	20	28	25	20.00	46.70	0.92	0.36	16.995
12	0.45	24	34	20	20.00	43.30	2.40	0.47	16.543
13	0.50	12	46	25	15.00	41.20	2.65	0.72	14.893
14	0.50	16	40	20	17.00	42.30	3.03	0.66	15.748
15	0.50	20	34	35	18.00	40.20	3.61	0.64	15.613
16	0.50	24	28	30	13.00	40.70	1.62	0.59	13.978

**Table 3:** Range analysis of experimental data.

**Factor**		**Initial Coagulation Time/min**						**Diffusion/mm**				
		**A**		**B**	**C**	**D**		**A**	**B**	**C**	**D**	
k1		22.250		22.2500	21	20.5		54.70	48.976	49.77	48.23	
k2		17.500		21.5000	19.75	20		50.42	50.35	47.80	48.08	
k3		22.750		17.7500	20.75	15		46.10	48.13	48.00	49.18	
k4		15.750		16.7500	16.75	22.75		41.10	44.86	46.75	46.85	
R		7		5.5	4.25	7.75		13.60	5.48	3.02	2.33	
**Factor**	**Compressive Strength/MPa**				**Shear Strength/MPa**				**Overall Rating**			
	**A**	**B**	**C**	**D**	**A**	**B**	**C**	**D**	**A**	**B**	**C**	**D**
k1	1.8235	1.9492	1.4332	2.3140	0.4303	0.5355	0.4405	0.5385	19.801	18.428	18.161	17.896
k2	1.330	1.980	2.1710	1.5532	0.5005	0.4980	0.5142	0.4938	17.438	18.582	17.559	17.532
k3	1.8660	2.0390	1.9855	1.8142	0.4572	0.5048	0.5053	0.4990	17.793	17.106	17.810	16.623
k4	2.7260	1.7773	2.1557	2.0640	0.6515	0.5013	0.5795	0.5082	15.057	15.972	16.559	18.043
R	1.3960	0.2617	0.7378	0.7607	0.2212	0.0375	0.139	0.0448	5.554	2.820	2.037	2.721

**Table 4:** Analysis of variance of initial setting time data.

Source of Variation	Sum of Squared Deviations	Degree of Freedom	Variance	*F*-Value	*Fα*	Significance Level
A	144.688	3	48.229		F0.01(3,15) = 5.417 F0.05(3,15) = 3.287 F0.10(3,15) = 2.490 F0.25(3,15) = 1.520	---
B	88.688	3	29.563			---
C	45.688	3	15.229			---
D	128.188	3	42.729			---
Error e	78.688	3	26.229			
Correct errors e	485.938	15	32.396			
Sum	485.938					

**Table 5:** Analysis of variance of diffusivity.

Source of Variation	Sum of Squared Deviations	Degree of Freedom	Variance	*F*-Value	*Fα*	Significance Level
A	93.847	3	31.282	3.324	F0.01(3,3) = 29.457 F0.05(3,3) = 9.277 F0.10(3,3) = 5.391 F0.25(3,3) = 2.356	o
B	205.445	3	68.482	7.278		*
C	96.573	3	32.191	3.421		o
D	82.922	3	27.641	2.937		o
Error e	28.229	3	9.410			
Sum	507.017					

* in the table indicates that this factor has some effect.

**Table 6:** Analysis of variance of compressive strength.

Source of Variation	Sum of Squared Deviations	Degree of Freedom	Variance	*F*-Value	*Fα*	Significance Level
A	4.036	3	1.3452	3.932	F0.01(3,12) = 5.953 F0.05(3,12) = 3.490 F0.10(3,12) = 2.606 F0.25(3,12) = 1.561	**
B	0.152	3	0.0505			---
C	1.434	3	0.4778			---
D	1.281	3	0.4270			---
Error e	1.240	3	0.4133			
Correct errors e	4.106	12	0.3422			
Sum	8.141					

** in the table indicates that the influence of this factor is more significant.

**Table 7:** Analysis of variance of shear strength.

Source of Variation	Sum of Squared Deviations	Degree of Freedom	Variance	*F*-Value	*Fα*	Significance Level
A	0.1170	3	0.0390	30.974	F0.01(3,9) = 6.992 F0.05(3,9) = 3.863 F0.10(3,9) = 2.813 F0.25(3,9) = 1.632	***
C	0.0040	3	0.0013			---
D	0.0388	3	0.0129	10.271		***
	0.0048	3	0.0016			---
Error e	0.0025	3	0.0008			
Correct errors e	0.0113	9	0.0013			
Sum	0.1670					

*** in the table indicates that the influence of this factor is very significan.

**Table 8:** Fitting relationship.

Factor	Index	Fitted Relationship	General Formula	Degree of Fit *R*^2^
Yellow mud quality Concentration (*P_A_*)	Initial coagulation time	$t_{1}=19.56+3.5\sin(20\pi P_{A}-0.5\pi )$	$t_{1}=t_{0}+A\sin\left(\pi \frac{P_{A}-x_{c}}{\omega }\right)$	0.92
	Diffusion	$K_{1}=24.477P_{A}{}^{-0.775}$	$K_{1}=aP_{A}{}^{b}$	0.98
	Compressive strength	$\sigma_{D1}=-940.67P_{A}{}^{3}+1334.7P_{A}{}^{2}-613.46P_{A}+93.36$	$\sigma_{D1}=aP_{A}{}^{3}+bP_{A}{}^{2}+cP_{A}+d$	0.99
	Tensile strength	$\sigma_{C1}=468P_{A}{}^{3}-584.3P_{A}{}^{2}+241.9P_{A}-32.724$	$\sigma_{C1}=aP_{A}{}^{3}+bP_{A}{}^{2}+cP_{A}+d$	0.99
High-water content (*P_B_*)	Initial coagulation time	$t_{2}=84.949P_{B}{}^{-0.511}$	$t_{2}=aP_{B}{}^{b}$	0.96
	Diffusion	$k_{2}=0.0067P_{B}{}^{3}-0.434P_{B}{}^{2}+8.536P_{B}-2.5$	$k_{2}=aP_{B}{}^{3}+bP_{B}{}^{2}+cP_{B}+d$	0.99
	Compressive strength	$\sigma_{D2}=-0.0026P_{B}{}^{3}+0.138P_{B}{}^{2}-2.381P_{B}+15.121$	$\sigma_{D2}=aP_{B}{}^{3}+bP_{B}{}^{2}+cP_{B}+d$	0.99
	Tensile strength	$\sigma_{C2}=1.96P_{B}{}^{3}-0.11P_{B}{}^{2}+0.186P_{B}-0.488$	$\sigma_{C2}=aP_{B}{}^{3}+bP_{B}{}^{2}+cP_{B}+d$	0.99
Cement content (*P_C_*)	Initial coagulation time	$t_{3}=-0.0056P_{C}{}^{3}+0.602P_{C}{}^{2}-21.345P_{C}+269.61$	$t_{3}=aP_{C}{}^{3}+bP_{C}{}^{2}+cP_{C}+d$	0.99
	Diffusion	$k_{3}=-0.0028P_{C}{}^{3}+0.316P_{C}{}^{2}-11.802P_{C}+194.262$	$k_{3}=aP_{C}{}^{3}+bP_{C}{}^{2}+cP_{C}+d$	0.99
	Compressive strength	$\sigma_{D3}=9.871P_{C}{}^{3}-0.114P_{C}{}^{2}+4.306P_{C}-51.81$	$\sigma_{D3}=aP_{C}{}^{3}+bP_{C}{}^{2}+cP_{C}+d$	0.99
	Tensile strength	$\sigma_{C3}=1.281P_{C}{}^{3}-0.014P_{C}{}^{2}+0.523P_{C}-5.875$	$\sigma_{C3}=aP_{C}{}^{3}+bP_{C}{}^{2}+cP_{C}+d$	0.99
Fly ash content (*P_D_*)	Initial coagulation time	$t_{4}=0.023P_{D}{}^{3}-1.815P_{D}{}^{2}+46.5P_{D}-367.5$	$t_{4}=aP_{D}{}^{3}+bP_{D}{}^{2}+cP_{D}+d$	0.99
	Diffusion	$k_{4}=-0.0062P_{D}{}^{3}+0.493P_{D}{}^{2}-12.69P_{D}+154.825$	$k_{4}=aP_{D}{}^{3}+bP_{D}{}^{2}+cP_{D}+d$	0.99
	Compressive strength	$\sigma_{D4}=-0.0014P_{D}{}^{3}+0.124P_{D}{}^{2}-3.62P_{D}+36.23$	$\sigma_{D4}=aP_{D}{}^{3}+bP_{D}{}^{2}+cP_{D}+d$	0.99
	Tensile strength	$\sigma_{C4}=-6.13P_{D}{}^{3}+0.0056P_{D}{}^{2}-0.167P_{D}+2.14$	$\sigma_{C4}=aP_{D}{}^{3}+bP_{D}{}^{2}+cP_{D}+d$	0.99

## Data Availability

The original contributions presented in the study are included in the article.
